# P79 Hypokalaemia—AmBisome versus Tillomed amphotericin

**DOI:** 10.1093/jacamr/dlag102.085

**Published:** 2026-06-26

**Authors:** Nazir S N Shazia

**Affiliations:** Leeds Teaching Hospitals NHS Trust, Leeds, UK

## Abstract

**Background:**

In December 2024, LTH transitioned from AmBisome to Tillomed amphotericin as part of a cost-saving initiative, with Tillomed selected as the lower-cost alternative. Following this change, internal observations suggested an increase in the number of patients developing hypokalaemia, including several severe cases and some associated with patient deaths. Concerns were particularly raised within adult haematology services regarding the incidence of hypokalaemia at a dosing regimen of 3 mg/kg with Tillomed. Hypokalaemia is a well-recognized adverse effect of amphotericin therapy, associated with renal potassium loss, and is highlighted in UK prescribing guidance such as the British National Formulary. Consequently, a review was undertaken to compare the incidence and clinical impact of hypokalaemia in patients treated with AmBisome versus Tillomed, and to assess whether the observed increase may be related to the change.

**Methods:**

A retrospective review was conducted of all patients prescribed AmBisome or Tillomed amphotericin from February 2024 onwards. The dataset was refined by removing duplicate prescriptions for the same patient and focusing specifically on those receiving a 3 mg/kg dosing regimen. Patients were identified using the electronic prescribing (eMeds) system, and records were reviewed to determine the occurrence of hypokalaemia. Data collected included treatment brand, patient age, duration of therapy, incidence and severity of hypokalaemia (classified as mild or severe based on potassium levels above or below 3.0 mmol/L), number of days potassium levels remained below the normal range, lowest recorded potassium level, time to onset of hypokalaemia, duration to recovery, and any interventions administered to correct hypokalaemia. In total, data from 134 prescriptions were analysed and recorded in a structured dataset for comparison.

**Results:**

A total of 65 prescriptions for patients receiving AmBisome 58% developed hypokalaemia, compared to 69 prescriptions for patients receiving Tillomed 59% developed hypokalaemia. Among these, 24 AmBisome patients and 30 Tillomed patients required treatment for hypokalaemia. It was noticed that patients in both groups had low potassium levels prior to initiation of therapy (11 in the AmBisome group and 23 in the Tillomed group), resulting in 25% patients already suffering from Hypokalaemia which may have affected them to further decline during treatment. Treatment discontinuation due to hypokalaemia occurred in 4 AmBisome patients (including 0 deaths) and 10 Tillomed patients (including 5 deaths).

**Conclusions:**

Although hypokalaemia rates were similar between groups, Tillomed was associated with higher rates of potassium replacement, treatment discontinuation, and mortality. However, many patients had low baseline potassium levels, and hypokalaemia is a recognized side effect of both treatments. These findings suggest that, while the choice of brand may play a role, patient-related factors such as baseline electrolyte status are also important contributors. This highlights the need for careful monitoring and optimization of electrolyte levels prior and maintenance therapy during amphotericin treatment alongside consideration of preventative strategies to minimize hypokalaemia and support continuation of treatment.

